# Beyond the Diagnostic Binary: A Translational Use of the Research Domain Criteria to Improve the Measurement of Anxiety in the Context of Cannabis Use

**DOI:** 10.3390/healthcare14152333

**Published:** 2026-08-01

**Authors:** Nicole Ennis, Katie Kloss, Alyssa Flores

**Affiliations:** Department of Behavioral Sciences & Social Medicine, Florida State University, Tallahassee, FL 32310, USA; katie.kloss@med.fsu.edu (K.K.); agf23@med.fsu.edu (A.F.)

**Keywords:** medical cannabis, recreational cannabis, anxiety, RDoC, mental health, substance use

## Abstract

**Highlights:**

**What are the main findings?**

- Anxiety is prevalent in both recreational and medical cannabis users with both groups endorsing clinically significant anxiety levels.
- Qualitative analyses revealed the anxiolytic and anxiogenic experiences of anxiety extended beyond traditional binary diagnostic categories and supports the use of the Research Domain Criteria (RDoC) framework as a valuable approach for understanding the relationship between anxiety and cannabis use.

**What is the implication of the main findings?**

- Future studies that examine cannabis use and anxiety should operationalize anxiety by focusing on the underlying biological and neurological mechanism rather than using a binary diagnostic category of symptom’s presence or absence.

**Abstract:**

Introduction: Epidemiological data indicate that over half of people who consume cannabis report anxiety reduction as a reason for use. Anxiety is frequently cited as a primary motivation for cannabis use among both medical and recreational consumers. The lifetime prevalence of anxiety disorders in the United States is approximately 34% with more recent data suggesting that the figure may be as high as 43%. Methods: Using a mixed methods design, we collected quantitative data through psychosocial assessment tools and demographic questionnaires from a sample of adult cannabis consumers (*n* = 30). These data were integrated with qualitative findings from semi structured interviews to contextualize consumers’ experiences and perceptions related to anxiety and cannabis use. Results: Medical cannabis consumers (*n* = 19) reported significantly more frequent consumption, averaging 28.95 days per month, as compared to recreational consumers (*n* = 11), averaging 23.55 days per month. Scores on the State-Trait Anxiety Inventory indicated clinically significant anxiety symptoms in both groups (39.95 for medical; 38.55 for recreational), with medical consumers more likely to endorse elevated anxiety. Qualitative analyses revealed nuanced and individualized interpretations of anxiety that extended beyond traditional binary diagnostic criteria revealing the Research Domain Criteria (RDoC) as a framework to better understand the relationship between anxiety and cannabis use. Discussion: The value of this work is its use of the RDoC framework to provide a new way to examine the relationship between anxiety and cannabis consumption. Currently the diagnosis of anxiety is binary based on DSM-5-TR criteria. This limitation makes it challenging to study the complex range of outcomes that occur when examining anxiety and cannabis use. Therefore, the opportunity to understand anxiety from a transdiagnostic framework such as RDoC enables researchers to study for whom and under what circumstances cannabis induces and/or reduces anxiety.

## 1. Introduction

Cannabis use is associated with a broad spectrum of biopsychosocial outcomes [1,2,3,4]. Current research shows that cannabinoids are important modulators of both the development and reduction in anxiety symptoms [5]. Therefore, it is imperative that we use a framework that accounts for symptomatology versus just presence or absence of anxiety to advance clinical science in this domain. Anxiety is frequently cited as a primary motivation for cannabis use among both medical [6,7,8,9,10] and recreational [11,12,13] consumers. The lifetime prevalence of anxiety disorders in the United States is approximately 34% [14] with more recent data suggesting that the figure may be as high as 43% [15]. However, among those who consume cannabis either medically or recreationally, anxiety occurs at a staggeringly higher rate [16]. Epidemiological data indicate that over half of people who consume cannabis report anxiety reduction as a reason for use [17], with up to 71.8% of medical cannabis consumers in one study reporting using cannabis in place of some or all anti-anxiety pharmaceuticals [18].

To date research on the relationship between anxiety and cannabis consumption indicates a more nuanced and complicated story. The experimental and clinical research literature shows that cannabis can be either anxiolytic or anxiogenic depending on the individual consumer [5,12,19]. The effects of cannabis on an individual’s anxiety depends on a host of factors including dose, the chemical makeup of the cannabis being consumed, and prior experience with cannabis [19,20]. Specifically, experimental and clinical research studies tend to find greater anxiogenic effects of cannabis use, suggesting cannabis use increases anxiety [19,21]. However, epidemiological data clearly show that individuals report using cannabis to reduce anxiety [7,12,17]. While there are several issues contributing to inconclusive results, one issue we identified for mixed and inconclusive results is how anxiety is operationalized. We posit that one reason for inconsistent results in experimental and clinical research in the context of cannabis use is due to the lack of standardized measurement that captures the complexity of how anxiety presents.

To that end, we identified the Research Domain Criteria (RDoC) as a more viable system for operationalizing anxiety in the context of cannabis use [22]. The RDoC framework conceptualizes psychopathology as dysfunctional variations along a spectrum of typical functioning with the goal of strengthening translational research. Instead of beginning with symptom clusters categorized as diagnoses in systems like the DSM or ICD and then searching for their biological basis, RDoC encourages researchers to first examine normal behavioral and neural processes and then investigate how disruptions in these systems may contribute to different symptoms and impairments. By emphasizing dimensions of functioning and observable behaviors rather than fixed diagnostic categories, RDoC seeks to address shortcomings in traditional diagnostic approaches to mental disorders.

The objective of this work is to apply the RDoC framework to operationalize anxiety in both medical and recreational cannabis consumers and examine whether anxiety presents differently in medical versus recreational cannabis consumers when starting from an adaptive understanding of anxiety using the RDoC framework. The RDoC framework’s focus on mechanisms supports researchers’ understanding of how anxiety develops. The framework looks across multiple levels of behavioral and physiological responses, and it looks at anxiety across a spectrum from adaptive to pathological. Therefore, this approach has the potential to support translational research and strengthen scientific rigor in this domain [23,24].

## 2. Materials and Methods

### 2.1. Study Aims

To improve our understanding of how anxiety presents in the context of cannabis use, the aims of the current study are to: (1) quantitatively and qualitatively describe self-reported symptoms of anxiety among medical and recreational cannabis consumers using the RDoC framework as comparted to the Diagnostic and Statistical Manual of Mental Disorders, Fifth Edition, Text Revision (DSM-5-TR); and (2) identify psychosocial and sociodemographic factors associated with use within each group.

### 2.2. Study Design

This study utilizes a mixed methods design leveraging quantitative measures to contextualize and describe the sample and qualitative methodology, specifically content analysis, to answer the research questions. In compliance with ethical standards, this protocol was approved by the Florida State University (FSU) Institutional Review Board (IRB approval #STUDY00001973). All procedures in this study were in accordance with the FSU Institutional Review Board’s ethical standard.

### 2.3. Study Participants

Participants include 30 adults (19 medical cannabis consumers and 11 recreational cannabis consumers) aged 18 to 69 who endorsed current medical and/or recreational cannabis use within the past 30 days. For the current study we used inductive thematic saturation, which relates to the qualitative data analysis process of ending data collection once no new codes have emerged from the data and only old ones are being repeated [25]. Participants were compensated $30 in cash for completion of a single session assessment. Assessments were conducted in person in our lab space at Florida State University or virtually via a HIPAA-compliant Zoom videoconferencing platform.

### 2.4. Recruitment

Medical cannabis participants were recruited via convenience sampling from the Medical Cannabis Treatment Clinics (MMTC) of Florida in Tallahassee using IRB-approved flyers posted in the clinic waiting areas. Recreational cannabis participants were similarly recruited in community settings such as local smoke shops, grocery stores, and coffee shops and through online forums such as Reddit using IRB-approved flyers.

### 2.5. Inclusion and Exclusion Criteria

A brief preliminary phone screen was conducted to assess basic eligibility criteria. Eligibility questions included: (a) Are you 18 years of age or older? and (b) Have you used medical and/or recreational cannabis in the past 30 days? Any individual who answered yes to both questions was eligible to be included in the study. After receiving a brief explanation of study procedures, individuals who remained interested in the study and met basic eligibility criteria were scheduled for a single timepoint assessment to complete both the quantitative and qualitative measures.

### 2.6. Data Collection Procedures

All data were collected during a single assessment session. Participants arrived for their appointments in person at our lab space within the Center for Translational and Behavioral Science at Florida State University (Tallahassee, FL, USA) or virtually via a HIPAA-compliant Zoom Version 5.12.2 videoconferencing platform. For participants interviewed in person, the informed consent was signed, scanned, and securely converted to a digital file stored in a password-protected drive on the FSU server. Participants who chose a Zoom appointment completed the informed consent via FSU’s HIPAA-compliant DocuSign platform, and the digital file was stored in the same manner.

After informed consent was obtained, data collection began. Participants provided a urine specimen for drug screening. A study kit with urinalysis cups and study description were mailed via FedEx to participants who chose the Zoom. Participants then completed a quantitative assessment battery via Research Electronic Data Capture (REDCap; Vanderbilt University City: Nashville, Tennessee, USA Version: 12.4), and a semi-structured interview using a structured interview guide (see Appendix A). Interviews averaged approximately 30–40 min in length. While a standardized guide was used, the interviews were semi-structured, allowing for further elicitation of emerging themes and/or clarification of existing themes. Interviews were conducted by the study PI and trained research assistants, digitally recorded, and professionally transcribed by an external HIPAA-compliant medical research transcription service. Transcriptions were spot-checked for accuracy; an error rate of greater than 5% prompted re-transcription of interviews. No transcription exceeded this error rate threshold. All audio recordings were stored on a password-protected server at FSU.

### 2.7. Study Measures

#### 2.7.1. Semi-Structured Interview Guide

A semi-structured interview guide was used to obtain qualitative data from participant responses to open-ended questions. The questions in the qualitative interview addressed cannabis use patterns, motivations for cannabis use, and perceived negatives and/or benefits that have resulted from cannabis use, and elicited what factors should be prioritized in cannabis research. See Appendix A for Structured Interview Guide.

#### 2.7.2. State-Trait Anxiety Inventory (STAI)

The STAI is a standard, reliable, and validated 40-item, two-part measure that quantifies self-reported anxious symptoms with the first 20 items assessing how the participant feels “right at the moment” of completing the survey (state), whereas the final 20 items assess how anxious the participant generally feels (trait) [26]. Participants rate all items of the STAI on a scale of 1 to 4, with 1 reflecting no endorsement of the item at all, and 4 indicating that the respondent “very much so” identifies with that item. A STAI cut score of 39–40 has been suggested to indicate clinically significant anxiety [27].

#### 2.7.3. Cannabis Use Disorder Identification Test—Revised (CUDIT-R)

The CUDIT-R is a standard, reliable, and validated 8-item measure assessing for the presence and severity of a cannabis use disorder (CUD) by assessing hazardous cannabis use, abuse, and dependence during the preceding six months [28]. Participants were instructed to answer each question on a scale from 0 to 4, with lower numbers indicating lower frequency of experiencing each item. Composite scores of at least 8 reflect potentially hazardous use of cannabis, while composite scores of at least 12 indicate the possibility of a CUD [28].

#### 2.7.4. Cannabis Use Questionnaire

The cannabis use questionnaire captured the participant’s age at first cannabis use, duration of cannabis use, frequency and amount of current cannabis use, routes of administration (both historical and current), and whether the participant has been recommended cannabis by a physician. Note: The term “recommended” is used in the state of Florida since medical cannabis prescriptions remain illegal federally.

#### 2.7.5. Sociodemographic Questionnaire

The sociodemographic questionnaire captured date of birth, sex, race, ethnicity, and socioeconomic status (via education, household income, insurance status, employment status).

#### 2.7.6. Center for Epidemiologic Studies Depression Scale (CES-D)

The CES-D is a standard, reliable, and validated 20-item depression screener that assesses how often the participant has experienced various self-reported depressive symptoms, including poor appetite, low mood, loneliness, and restless sleep [29]. Participants respond to each item quantifying how often they felt or behaved in that manner during the past week with options including “rarely or none of the time (less than 1 day),” “some or a little of the time (1–2 days),” “occasionally or a moderate amount of the time (3–4 days),” and “most or all of the time (5–7 days).” CES-D scores above the cut score of ≥16 indicate possible risk for clinical depression [29].

#### 2.7.7. Urinalysis

A commercially available urine drug test kit was used to biologically confirm cannabis use for all participants [30].

### 2.8. Data Analysis

#### 2.8.1. Quantitative Analysis

We computed descriptive statistics to provide sociodemographic and clinical characterization of the study sample. We examined whether the medical and recreational participants differed at the time of assessment on demographic and clinical variables of interest using chi-square for binary variables and *t*-test for continuous variables. The goal of the current work is to qualitatively describe cannabis use and self-reported anxiety, in addition to, motivations for and perceptions of current cannabis use. The descriptive statistics are used to contextualize the sample.

#### 2.8.2. Qualitative Analysis: Content Analysis

**Figure 1:**
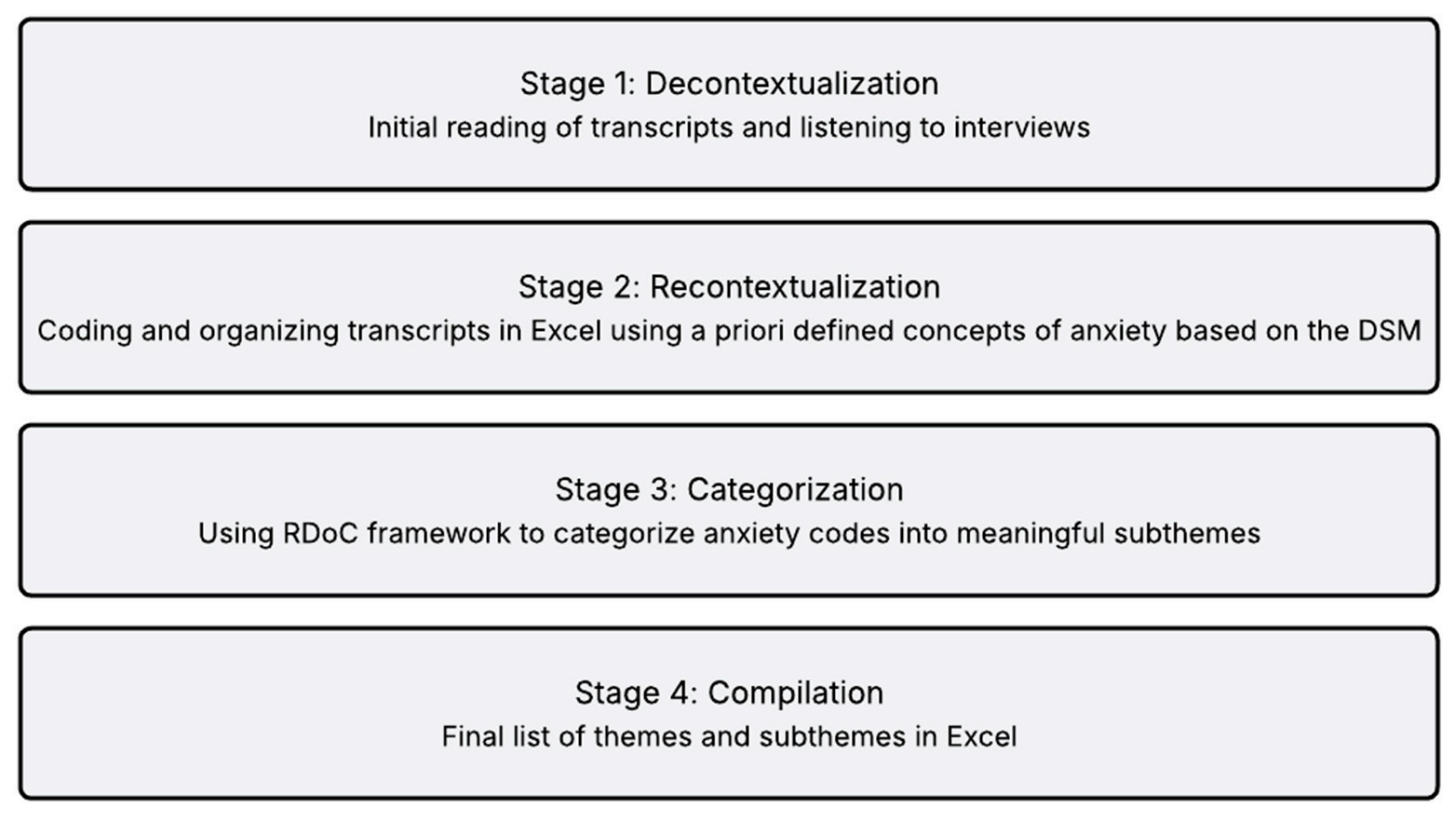
Content analysis framework.

Qualitative analyses were conducted using the content analysis framework proposed by Bengtsson [31], which lays out four stages to the conduct of qualitative analyses. Stage 1 is the decontextualization, during which the study team read through the transcript and listened to the associated audio file. In stage 2, recontextualization, we isolated all text in the transcript that was associated with anxiety, organized them in an Excel spreadsheet organized by participant number, and identified meaningful units of analysis by developing and defining anxiety using the standard DSM-5-TR diagnostic framework [32]. In stage 3, categorization, subthemes were identified. Using the RDoC framework [22] we categorized all the anxiety codes into six domains (i.e., negative valence systems, positive valence systems, cognitive systems, social processes, arousal and regulatory systems, and sensorimotor systems) to categorize the meaningful units identified in stage 2. In stage 4, compilation, we used manifest analysis (i.e., using the participant’s words) to identify and characterize the magnitude of the phenomena identified. See Figure 1.

A deductive content analysis approach, which is a “top-down” approach to data analysis, was used for this study. Content analysis is a research methodology that allows for the systematic and objective means of describing and quantifying individual experiences, allowing the research team to test theoretical issues that will enhance our understanding of the experiences of interest. Deductive content analysis involves applying theory (in this case the RDoC translational framework intended to reframe mental health research using biological and behavioral factors rather than traditional symptom-based diagnostic categories [22,33]) to the data to test how well the data fit the theory. We examined anxiety as defined by the DSM-5-TR—a diagnostic approach [32]—and then subcategorized the data using the RDoC framework [22]. With the structure provided by our established research questions, we defined self-reported anxiety using the DSM-5-TR. We organized these definitions into a table for ease of reference when completing content analysis and used an Excel spreadsheet to document when and where any self-reported anxiety meeting the DSM-5-TR definition was identified using the code AX. Field notes were taken and maintained throughout our content analysis to identify any potential bias. Coding was completed by two research assistants, and consensus coding with the PI was used to resolve all conflicts and arrive at the final codes.

In this mixed methods study, quantitative and qualitative data were integrated during the interpretation phase to provide a more comprehensive understanding of the findings. Quantitative results were used to contextualize the qualitative themes by identifying broader patterns and trends within the sample, while the qualitative data offered deeper insight into participants’ experiences and perspectives. The convergence of findings across both data sources strengthened the credibility of the results, with quantitative data serving to validate and support the interpretations derived from the qualitative analysis.

### 2.9. Generative AI

Artificial intelligence (AI) tools were used to assist with improving the clarity, readability, and overall flow of the manuscript, while all substantive content, interpretation, and final revisions remained the responsibility of the authors. See Appendix B for detailed AI disclosure.

## 3. Results

### 3.1. Quantitative

#### 3.1.1. Sample Characteristics

The total study sample (N = 30) consisted of both medical (*n* = 19) and recreational (*n* = 11) cannabis consumers. In the overall sample, 63.33% were male, 83.33% were white, and the sample ranged in age from 18 to 69 with an average of 35.33 years and a standard deviation of 14.18 years. Nearly half of participants reported attending community college, technical school, or “some college” (43.33% combined), with 30% reporting being college graduates and 13.33% reporting engagement in post-graduate studies. Most of the sample reported having either full-time or part-time employment (46.67% and 20%, respectively), and 86.67% reported having health insurance. See Table 1.

#### 3.1.2. Sociodemographics Stratified by Cannabis Use Group

Frequency distributions for sample sociodemographics were also analyzed separately for each group, as presented in Table 1. The medical cannabis group was older with an average of 38.11 years of age (SD 14.03) at the time of assessment, compared to an average of 30.55 (SD 13.75) years of age for the recreational group. Although both samples had a majority of white participants (89.50% for medical and 72.70% for recreational), the recreational cannabis sample was slightly more racially diverse with 18.20% of participants being black/African American. Males make up the majority of the sample in both groups (63.20% for medical and 63.60% for recreational). Both the medical and recreational cannabis use samples primarily reported attending some college or being a college graduate (47.40%, 31.60% and 36.40%, 27.30% respectively). The majority of recreational consumers reported either full-time or part-time employment (54.50% and 36.40% respectively), while the medical group was more varied with some participants reporting disability, retirement, and unemployment (15.80%, 5.30%, and 15.80%, respectively).

#### 3.1.3. Cannabis Use Patterns

The overall study sample’s average age of first recreational cannabis use was 16.27 (SD 3.59) years old, and participants reported an average of 14.17 (SD 14.95) years of regular cannabis use with an average of 26.97 (SD 6.10) reported days of use in the past 30 days. In total, 63.33% of the sample confirmed that in the past month cannabis had been recommend by a physician and, as a result, comprised the medical cannabis group. In the past 30 days, a majority of the overall sample reported using vapes/electronic devices (63.33%) and edibles/potables (60%) as routes of administration for cannabis.

#### 3.1.4. Cannabis Use Patterns Stratified by Cannabis Use Group

Frequency distributions for cannabis use patterns were stratified for each group in Table 2. More medical consumers reported using vaping/electronic devices (68.40%) and edibles/potables (68.40%) to consume cannabis in the past 30 days, whereas more recreational consumers reported consuming cannabis by smoking joints and blunts (63.60%). Participants in the medical group had more cannabis use days in the past 30 days with an average of 28.95 days (SD 2.15) as compared to recreational consumers who consumed an average of 23.55 days (SD 8.90).

#### 3.1.5. Scores on Psychosocial Measures

Participants completed standardized psychosocial measures (i.e., CUDIT-R, STAI) to assess for the possible presence of a CUD and anxiety symptoms (See Table 3). The study sample scored an average of 14.33 (SD 5.46) on the CUDIT-R, indicating symptoms consistent with a possible CUD with 43.33% of the sample above the cut score of ≥12. The study sample had an average STAI state score of 34.43 and a STAI trait score of 39.43. A STAI cut score of 39–40 has been suggested to indicate clinically significant anxiety [27].

#### 3.1.6. Scores on Psychosocial Measures Stratified by Cannabis Use Group

The scores from the screening tools (CUDIT-R and STAI), which were also calculated to compare the presence of a CUD and anxiety symptoms in both groups, are presented in Table 3. Both the medical and recreational groups scored with symptoms indicating a possible CUD diagnosis (14.26/SD 5.34 and 14.45/SD 5.92 respectively). Neither group had statistically significant differences in their STAI state score (35.26 for medical and 33 for recreational) or their STAI trait score (39.95 and 38.55, respectively). However, the trait scores approached clinical significance.

### 3.2. Qualitative

#### 3.2.1. Negative Valence Systems

Negative Valence Systems as defined by RDoC are responsible for “responses to aversive situations or context, such as fear, anxiety, and loss [34].” The RDoC framework lists the following constructs associated with this subcode: Acute Threat (“Fear”), Potential Threat (“Anxiety”), Sustained Threat, Loss, and Frustrative Nonreward. Overall, 13 negative valence codes were identified for all participants. The negative valence codes were 33% of all codes, the largest number found. Medical and recreational codes did not differ in percentage. See Table 4 and Table 5.

#### 3.2.2. Positive Valence Systems

Positive Valence Systems as defined by RDoC are primarily responsible for “responses to positive motivational situations or contexts, such as reward seeking, consummatory behavior, and reward/habit learning [34].” The RDoC framework lists the following associated constructs: Reward Responsiveness, Rewards Learning, and Reward Valuation. Two codes were identified as positive valence systems among all the participants, making up only 5% of all codes. This subcode was only identified in the medical group. See Table 4 and Table 5.

#### 3.2.3. Cognitive Systems

Cognitive systems as defined by RDoC are responsible for various cognitive processes, such as Attention, Perception, Declarative Memory, Language, Cognitive Control, and Working Memory [34]. Cognitive systems make up 15.38% of all codes with 3 relevant codes identified in each group. See Table 4 and Table 5.

#### 3.2.4. Social Processes

The social processing system as defined by RDoC is responsible for “mediating a variety of interpersonal responses [34].” The following constructs comprise this subcode: Affiliation and Attachment, Social Communication, Perception and Understanding of Self, and Perception and Understanding of Others [34]. Eight relevant codes (5 for medical, 3 for recreational) were identified, which made up 21% of all codes across the study sample. See Table 4 and Table 5.

#### 3.2.5. Arousal and Regulatory Systems

Arousal and Regulatory Systems as defined by RDoC are responsible for “generating activation of neural systems as appropriate for various contexts and providing appropriate homeostatic regulation of such systems as energy balance and sleep [34].” They are made up of the following constructs: Arousal, Circadian Rhythms, and Sleep-Wakefulness [34]. Overall, 10 codes were identified from the overall sample, making up 26% of all codes. More relevant codes were identified for this domain in the medical cannabis group (30%) than the recreational cannabis group (17%). See Table 4 and Table 5.

#### 3.2.6. Sensorimotor Systems

Sensorimotor Systems as defined by RDoC are primarily responsible for the “control and execution of motor behaviors, and their refinement during learning and development [34].” The RDoC matrix describes the following related constructs: Motor Actions, Agency and Ownership, Habit-Sensorimotor, and Innate Motor Patterns [34]. There were no relevant codes identified from the study sample. See Table 4 and Table 5.

## 4. Discussion

National demographic trends suggest that medical cannabis consumers are more likely to be older, white, male, and have higher levels of education [26,27,28,29]. In the current study, most participants in both groups identified as white (89.5% of medical consumers and 72.7% of recreational consumers) and male (63.2% and 63.6%, respectively). However, the recreational group included two participants who identified as black/African American, and both groups included an equal number of Hispanic participants. Although prior research generally indicates that medical cannabis consumers report higher incomes, recreational participants in this study reported significantly greater monthly income than medical participants. This finding should be interpreted cautiously due to the high standard deviation for income in the recreational group, suggesting substantial variability influenced by an outlier within the small sample. Employment status also differed between groups. Recreational participants primarily reported full-time or part-time employment, with one participant identifying as a student, whereas the medical group demonstrated greater variability, including individuals who were disabled, retired, or unemployed. Overall, these findings align with several national demographic trends; however, Florida-specific data indicate equal or greater representation of female medical cannabis consumers, and comparable demographic data for recreational consumers remain limited due to privacy-related restrictions [9,35].

Medical cannabis patients’ motivations for use often reflect the symptoms prevalent in this population, such as sleeplessness and anxiety [36]. In the first stage of qualitative coding we found that a greater proportion of medical cannabis consumers in our sample reported anxiety symptoms, as defined by the DSM-5-TR as compared to recreational consumers. However, both groups received an average STAI trait score that indicated clinical anxiety, and neither group had statistically significant differences in scoring. Comparatively, both average STAI state scores were below the cut-off, which we posture is a result of the welcoming, low-pressure environment of the study questionnaire [37]. Extant literature also indicates that medical cannabis use may increase the likelihood of possible CUD [36,38]. Our research suggests that the medical group consumed cannabis more frequently than the recreational group in the 30 days prior to their assessment. However, this may be due to greater, more consistent access to legally purchase cannabis products at local dispensaries. Frequency of use has been associated with a greater likelihood of developing a CUD [39,40]. While there was no significant difference in CUDIT-R scores between the two groups, both groups’ averages were above the cut-off score of ≥12.

The most common ROAs differed between medical consumers and recreational consumers with recreational consumers reporting the use of blunts and joints as their primary ROA. However, medical cannabis patients in the state of Florida have legal access to a variety of routes of administration, including sublingual, topical, inhalation, edible, and oral that are not currently as readily available to recreational consumers. Therefore, similar to frequency of use, the variability in routes of administration may be attributed to greater accessibility through local dispensaries. Most participants in the medical group (68.4%) reported consuming cannabis for both medical and recreational reasons, whereas three participants (27.3%) in the recreational group reported consuming for medical reasons only. More research is needed with a larger sample to discern distinctions between the two groups. However, this may suggest that cannabis consumption for medical and recreational purposes can be found in both groups.

The value of this work is its use of the RDoC framework to provide a new way to examine the relationship between anxiety and cannabis consumption. Currently anxiety disorders are diagnosed as present or absent based on DSM-5-TR criteria. This limitation makes it challenging to study the complex range of outcomes that occur when examining anxiety and cannabis use. Therefore, the opportunity to understand anxiety from a transdiagnostic framework such as RDoC enables researchers to study for whom and under what circumstances cannabis induces and/or reduces anxiety. Our use of the DSM-5-TR to first identify and capture all self-reported anxiety in relation to cannabis consumption, and then subcategorize those responses into the RDoC framework for a clear sense of what factors and systems are driving the relationship between anxiety and cannabis consumption, enables researchers to move forward the science in this field. This approach supports testing questions that examine both how cannabis induces anxiety or extinguishes anxiety depending on what system is activated. RDoC was initiated specifically to find new ways to examine mental health and clinically relevant processes that have a neurobiological as well as psychological referent such as anxiety. RDoC has allowed researchers to establish distinct psychopathologies for anxiety condition involving generalized distress and dysphoria versus those involving focal fear [33]. This is particularly important for anxiety disorders such as PTSD where the diagnostic binary of anxiety addresses numerous arousal symptoms that need to be understood separately. For example, negative valance symptoms such as fear are separate from autonomic disturbances that lie in the domain of arousal/regulatory systems. The RDoC framework allowed us to analyze anxiety without neglecting the nuances and layers that make up each participant’s experience with the disorder. The majority of participants’ self-reported anxiety symptoms were coded as “negative valence systems” and “arousal and regulatory systems.” Our medical group made up most of the “ARS” codes, which involves arousal and sleep-wakefulness. Considering that many medical cannabis consumers medicate for PTSD symptoms and sleep, it was not surprising to find this distinction between our groups [41,42].

Noteworthy strengths of this study were our mixed methods approach and the use of a rigorous content analysis methodology, which enabled us to open a new direction for understanding the relationship between the variables of interest (anxiety and cannabis consumption). Another strength is the innovation regarding the issue of how to study cannabis consumption in a rigorous manner given its paradoxical outcomes on anxiety. Our work highlights new directions for research in this field by leveraging the RDoC transdiagnostic framework. While innovative, our study had a few limitations. First, the sample size was small, and while it is within range for qualitative studies, it does not give us the power needed to examine differences between groups. Furthermore, the recreational cannabis group was substantively smaller than the medical group, which is likely a result of the potential legal implications of recreational cannabis use, as Florida is not currently a recreational use state. Therefore, the recreational consumers were a hard-to-reach sample. This also suggests that the population of recreational consumers willing to participate may be different from those we could not reach. Therefore, another limitation is generalizability. Since we used convenience sampling, our sample may not be representative of all medical and recreational cannabis consumers.

## 5. Conclusions

Our study sheds light on the distinct characteristics and consumption patterns between medical and recreational cannabis consumers, as well as the challenges in disentangling the two. It also provides a vital way forward to study cannabis consumption in the context of anxiety. No other study to date has used the translational framework by the National Institute of Mental Health to understand and disentangle participants’ experience of cannabis consumption and self-reported anxiety. Despite limitations in sample size, our findings highlight the prevalence of anxiety symptoms among both groups and the potential role of cannabis in symptom management. Our use of the RDoC framework provides a nuanced examination of anxiety symptoms and individual experiences with cannabis in our study sample. Larger studies are needed to validate and expand our research to broaden the current understanding of cannabis consumption and anxiety.

## Figures and Tables

**Table 1:** Sample sociodemographics by group (*N* = 30).

Variable	Entire Sample *n* (%)/Mean (SD)	Medical *n* (%)/Mean (SD)	Recreational *n* (%)/Mean (SD)	Test Statistics *χ*^2^ or *t*-Test *p*-Value	
N	30	19	11		
Age	35.33 (14.18)	38.11 (14.03)	30.55 (13.75)	0.047	0.83
Race				0.093	0.763
White	25 (83.33)	17 (89.50)	8 (72.70)		
Black/African American	2 (6.67)	0 (0.00)	2 (18.20)		
Other	3 (10.00)	2 (10.50)	1 (9.10)		
Ethnicity				0.036	0.85
Hispanic/Latino	6 (20.00)	4 (21/10)	2 (18.20)		
Sex				0.627	0.731
Male	19 (63.33)	12 (63.20)	7 (63.60)		
Female	10 (33.33)	6 (31.60)	4 (36.40)		
Prefer not to say	1 (3.33)	1 (5.30)	0 (0.00)		
Education				0.457	0.504
High school or GED	4 (13.33)	2 (10.50)	2 (18.20)		
Junior/Tech/Some college	13 (43.33)	9 (47.40)	4 (36.40)		
College graduate	9 (30.00)	6 (31.60)	3 (27.30)		
Post-college/graduate	4 (13.33)	2 (10.50)	2 (18.20)		
Monthly income (USD)	5803 (10,193)	4544 (4294)	7977 (16,121)	4.918	0.035 *
Employment status				4.96	0.034 *
Full-time	14 (46.67)	8 (42.10)	6 (54.50)		
Part-time	6 (20.00)	2 (10.50)	4 (36.40)		
Unemployed	3 (10.00)	3 (15.80)	0 (0.00)		
Retired	1 (3.33)	1 (5.30)	0 (0.00)		
Disabled	3 (10.00)	2 (15.80)	0 (0.00)		
Student	3 (10.00)	2 (10.50)	1 (9.10)		
Health insurance status				0.271	0.603
Yes	26 (86.67)	16 (84.20)	10 (90.90)		
No	4 (13.33)	3 (15.80)	1 (9.10)		

* indicate statistical significance.

**Table 2:** Cannabis use patterns by group (*N* = 30).

Variable	Entire Sample *n* (%)/Mean (SD)	Medical *n* (%)/Mean (SD)	Recreational *n* (%)/Mean (SD)
N	30	19	11
Age of first recreational cannabis use	16.27 (3.59)	16.37 (4.36)	16.09 (1.76)
Years of regular cannabis use	14.17 (14.95)	15.74 (14.95)	11.45 (15.28)
Ways used cannabis (lifetime)			
Joint	29 (96.67)	18 (94.70)	11 (100.00)
Bowl/pipe	27 (90.00)	17 (89.50)	10 (90.90)
Blunt	25 (83.33)	15 (78.90)	10 (90.90)
Vaping/electronic device	27 (90.00)	17 (89.50)	10 (90.90)
Hookah/bong/waterpipe	25 (83.33)	16 (84.20)	9 (81.80)
Baked/drinking	29 (96.67)	18 (94.70)	11 (100.00)
Other	13 (43.33)	11 (57.90)	2 (18.20)
Recommended medical cannabis (lifetime)			
Yes	19 (63.33)	19 (100.00)	0 (0.00)
No	11 (36.67)	0 (0.00)	11 (100.00)
Used cannabis (past 30 days)			
Yes	30 (100.00)	19 (100.00)	11 (100.00)
No	0 (0.00)	0 (0.00)	0 (0.00)
Ways used cannabis (past 30 days)			
Joint	16 (53.33)	9 (47.40)	7 (63.60)
Bowl/pipe	13 (43.33)	9 (47.40)	4 (36.40)
Blunt	11 (36.67)	4 (21.10)	7 (63.60)
Vaping/electronic device	19 (63.33)	13 (68.40)	6 (54.50)
Hookah/bong/waterpipe	16 (53.55)	10 (52.60)	6 (54.50)
Baked/drinking	18 (60.00)	13 (68.40)	5 (45.50)
Other	4 (13.33)	4 (21.10)	0 (0.00)
Has any past month cannabis use been recommended by a physician?			
Yes	19 (63.33)	19 (100.00)	0 (0.00)
No	11 (36.67)	0 (0.00)	11 (100.00)
How many days in the past 30 days have you used cannabis?	26.97 (6.10)	28.95 (2.15)	23.55 (8.90)
In the past 30 days, have you used cannabis for medical or non-medical purposes?			
Only for medical purposes	8 (26.67)	5 (26.30)	3 (27.30)
Only for non-medical purposes	3 (10.00)	1 (5.30)	2 (18.20)
Both	17 (56.67)	13 (68.40)	4 (36.50)
Unsure	2 (6.67)	0 (0.00)	2 (18.20)

**Table 3:** Cannabis use disorder and state and trait anxiety by group (*N* = 30).

Variable	Entire Sample *n* (%)/Mean (SD)	Medical *n* (%)/Mean (SD)	Recreational *n* (%)/Mean (SD)	Test Statistics *χ*^2^ or *t*-Test *p*-Value	
N	30	19	11		
CUDIT-R				0.004	0.948
Total Score	14.33 (5.46)	14.26 (5.34)	14.45 (5.92)		
<12	17 (56.67)	8 (42.1)	5 (45.5)		
≥12 (CUD)	13 (43.33)	11 (57.9)	6 (54.5)		
CES-D				0.821	0.373
Total Score	14.03 (11.46)	13.68 (11.28)	14.64 (12.30)		
<16	24 (80.00)				
≥16	6 (20.00)				
STAI State (Y1)	34.43 (10.12)	35.26 (8.81)	33 (12.39)	0.734	0.399
STAI Trait (Y2)	39.43 (10.42)	39.95 (9.80)	38.55 (11.86)	0.442	0.512

**Table 4:** DSM-5-TR codes identified for each RDoC construct by group.

RDoC Constructs	DSM-5-TR Codes Identified for Each Construct Entire Sample *n* (%)	DSM-5-TR Codes Identified for Each Construct Medical *n* (%)	DSM-5-TR Codes Identified for Each Construct Recreational *n* (%)
Negative Valence Systems	13 (33.33)	9 (33.33)	4 (33.33)
Positive Valence Systems	2 (5.13)	2 (7.74)	0 (0.00)
Cognitive Systems	6 (15.38)	3 (11.11)	3 (25.00)
Social Processes	8 (20.51)	5 (18.51)	3 (25.00)
Arousal and Regulatory Systems	10 (25.64)	8 (29.63)	2 (16.66)
Sensorimotor Systems	0 (0.00)	0 (0.00)	0 (0.00)
Total	39	27	12

**Table 5:** Content analysis coding example quotes for each RDoC construct.

RDoC Construct	Quote	Age	Sex	Group (Participant ID)
Negative Valence Systems	*“I suffer from anxiety. It’s not crippling but it does get in the way of […] my studies or work or, you know, personal life, personal issues.”*	24	Male	Medical (1008)
	*“If I’m real stressed out or anxious over something or almost have like an anxiety or panic attack, I can smoke.”*	35	Male	Medical (1017)
Positive Valence Systems	*“ I think it also calms my nerves a little bit. […] my mental health has been pretty good, but in the past when it was a little worse […] it was a little bit of a help there.”*	23	Male	Recreational (1019)
	*“[Consuming cannabis] like calms my nerves. I guess anxiety reducing.”*	22	Female	Medical (1014)
Cognitive Systems	*“…I think mostly it’s like internal consciousness […] my internal rhetoric, my thoughts, and stuff like that. And then a little bit of calming and relief of anxiety. I guess I’m just learning that I really do have anxiety. I’ve been smoking for like five years, but um, I didn’t really learn about anxiety or that it was real because I was raised in kind of a military environment. So, it was very like hey just like, you know, you don’t have any problems. You know what I mean. And so now, I’m definitely, I’m definitely right. It’s like that’s what I’m, that’s what I’ve been like medicating for.”*	24	Male	Medical (1020)
	*“[Cannabis] also helps with anxiety and relaxation […] I do use it also for racing thoughts.”*	31	Female	Recreational (1022)
Social Processes	*“It helps me be more present and not like have the anxieties of, oh, I’m not doing this right now, I should be, you know […] I did have a bit of a social anxiety so I did start off my recreational use like that because also the anxious thoughts like, oh, these people are laughing because of what we said. When I’m smoking, I’m just like ah, we’re all just laughing, we’re having a good time, whatever.”*	22	Female	Recreational (1009)
	*“My reasons for using [cannabis] are […] general anxiety, social anxiety, things of that nature.”*	39	Male	Medical (1023)
Arousal and Regulatory Systems	*“…the edibles are new. I have not been able to really put a handle on it. I do not do them every day, but I will do them. I will do a 10 milligram [edible] and I can sleep better. I have always had a hard time sleeping. It is hard for me to shut my mind off. When I wake up to go pee, I cannot go back to sleep. It is a never-ending saga. I have things to keep me busy until I do decide to go back to sleep. Pot would help. If I did 20 milligrams, I can probably sleep all night without a problem. It would really help.”*	65	Male	Medical (1005)
	*“I decided to stop taking Adderall and that kind of dysregulated my [sleep], appetite, and weed also helped me deal with that too.“*	39	Male	Medical (1023)
Sensorimotor Systems	No codes identified in this category.	n/a	n/a	n/a

**Figure A1:**
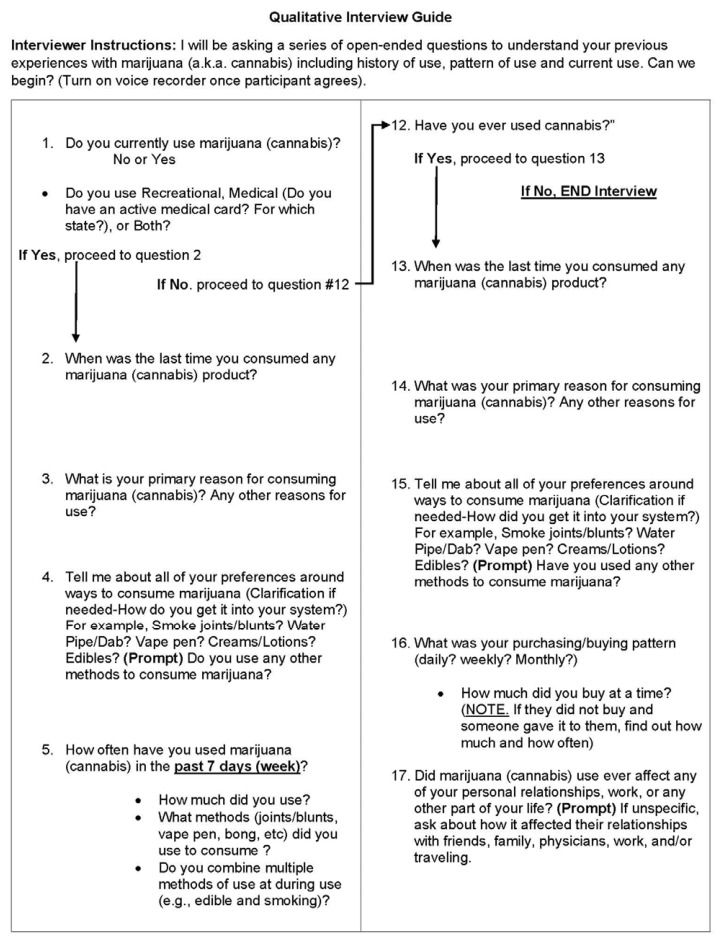
Structured interview guide (page 1).

**Figure A2:**
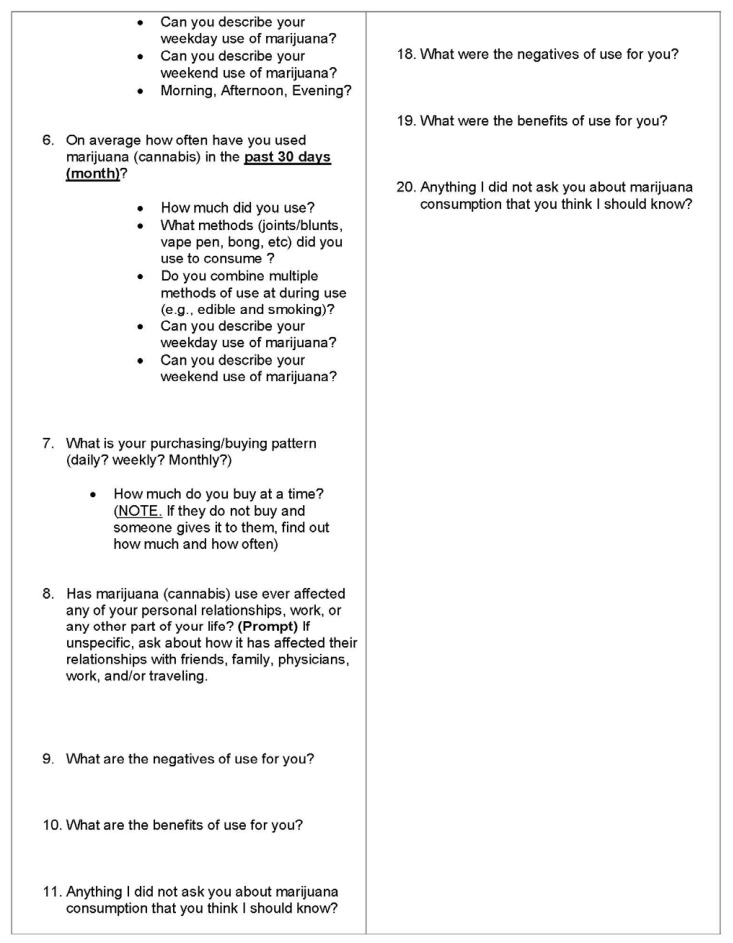
Structured interview guide (page 2).

**Figure A3:**
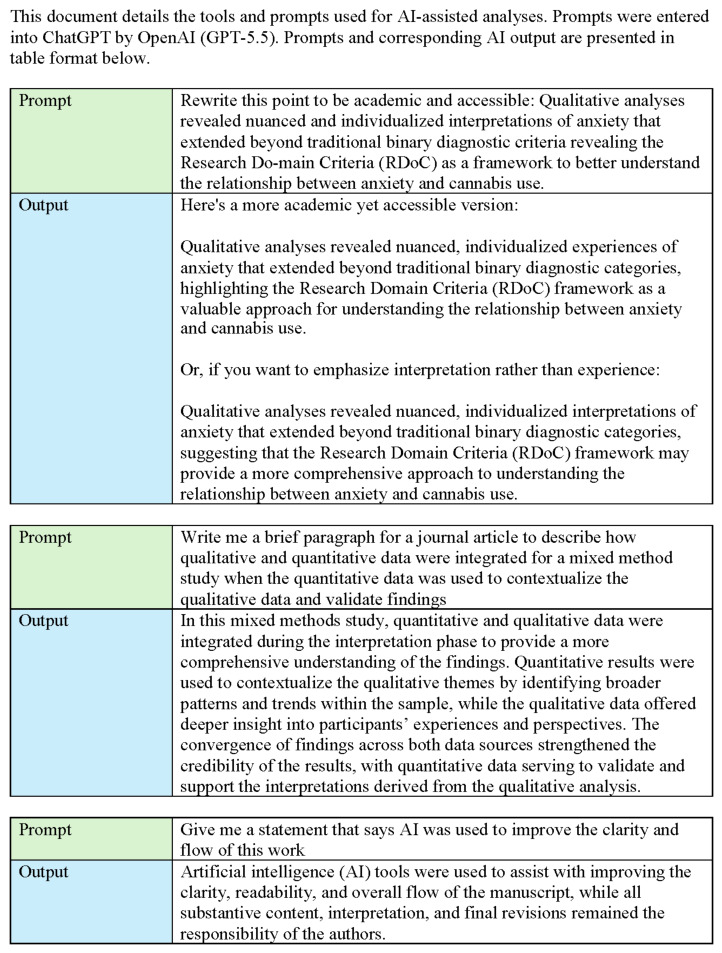
Artificial intelligence disclosure (page 1).

**Figure A4:**
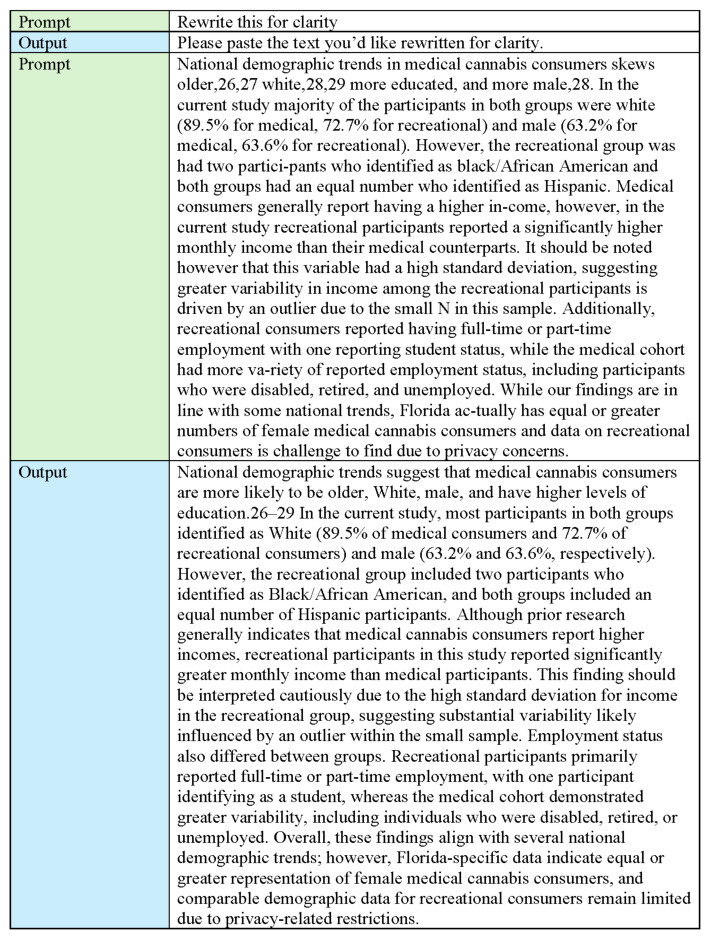
Artificial intelligence disclosure (page 2).

**Figure A5:**
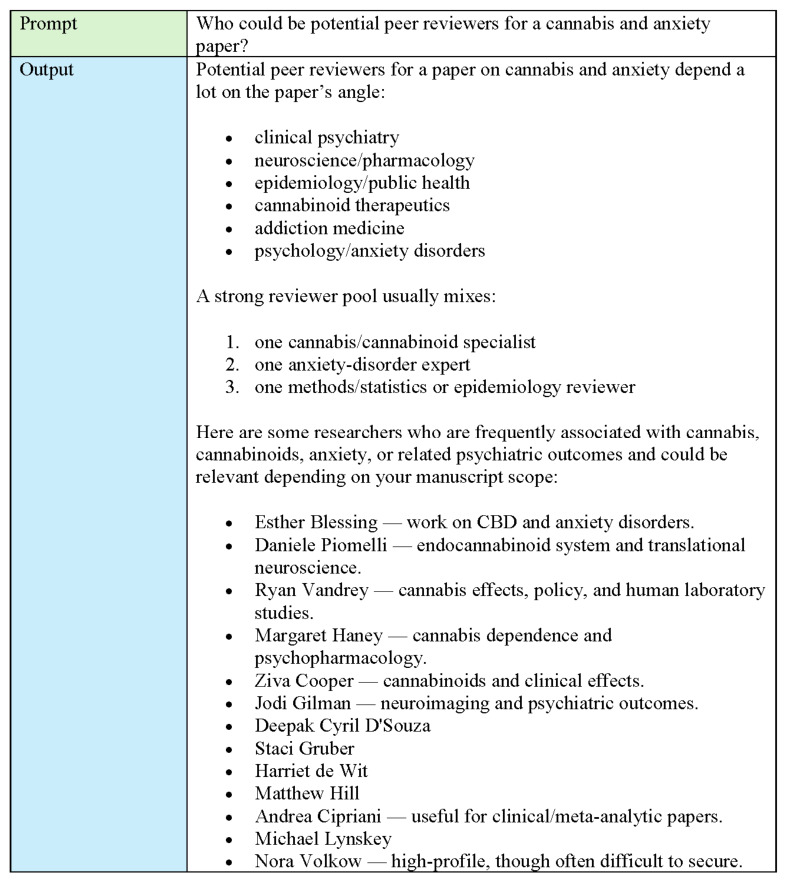
Artificial intelligence disclosure (page 3).

## Data Availability

For external parties, de-identified data, presented in aggregate form, will be made available upon request from the study’s lead author, with adequate ethical approval from the relevant home institution.

## Abbreviations

The following abbreviations are used in this manuscript:

CES-D	Center for Epidemiologic Studies Depression Scale
CUD	Cannabis Use Disorder
CUDIT-R	Cannabis Use Disorder Identification Test-Revised
DSM-5-TR	Diagnostic and Statistical Manual of Mental Disorders, Fifth Edition, Text Revision
FSU	Florida State University
ICD	International Classification of Diseases
IRB	Institutional Review Board
MMTC	Medical Cannabis Treatment Clinics of Florida
PI	Principal Investigator
RDoC	Research Domain Criteria
ROA	Route of Administration
STAI	State-Trait Anxiety Inventory
